# Alterations in Blood Plasma Metabolome of Patients with Lesniowski-Crohn’s Disease Shortly after Surgical Treatment—Pilot Study

**DOI:** 10.3390/metabo12060529

**Published:** 2022-06-08

**Authors:** Jakub Idkowiak, Grażyna Kubiak-Tomaszewska, Paulina Gątarek, Łukasz Marczak, Joanna Kałużna-Czaplińska, Wiesław Tarnowski, Mariusz Uryszek, Barbara Bobrowska-Korczak

**Affiliations:** 1 Department of Bromatology, Faculty of Pharmacy, Medical University of Warsaw, Stefana Banacha 1, 02-097 Warsaw, Poland; jakubidkowiak1@gmail.com (J.I.); barbara.bobrowska@wum.edu.pl (B.B.-K.); 2 Department of Analytical Chemistry, Faculty of Chemical Technology, University of Pardubice, Studentská 573, CZ-532 10 Pardubice, Czech Republic; 3 Department of Biochemistry and Clinical Chemistry, Faculty of Pharmacy, Medical University of Warsaw, Stefana Banacha 1, 02-097 Warsaw, Poland; grazyna.kubiak-tomaszewska@wum.edu.pl; 4 Department of Chemistry, Institute of General and Ecological Chemistry, Lodz University of Technology, Stefana Żeromskiego 116, 90-924 Lodz, Poland; gatarekpaulina@gmail.com; 5 Institute of Bioorganic Chemistry, Polish Academy of Sciences, Zygmunta Noskowskiego 12/14, 61-704 Poznan, Poland; lukasmar@ibch.poznan.pl; 6 Department of General, Oncological and Digestive Tract Surgery, Medical Centre of Postgraduate Education, Orłowski Hospital, 00-416 Warsaw, Poland; tarnowskiwieslaw@me.com (W.T.); mariusz_ury@mac.com (M.U.)

**Keywords:** Gas Chromatography-Mass Spectrometry (GC-MS), metabolomics, blood plasma metabolome, Lesniowski-Crohn’s disease

## Abstract

Lesniowski-Crohn’s disease (CD) is a type of chronic inflammatory bowel disease (IBD) of uncertain etiology. Initially, pharmacological management is undertaken; however, surgical intervention is necessary to improve life quality and relieve symptoms in most cases. Here changes are reported in blood metabolome that occurred three days after the ileo-colic region resection in the case of seven patients. Alterations are observed in levels of metabolites associated with multiple mitochondrial pathways, based on the Metabolite Set Enrichment Analysis, reflecting a high energy demand in the post-operative period. As most of these metabolites are also essential nutrients supplied from foods, we believe that our results might contribute to the discussion on perioperative nutrition’s role in enhanced recovery.

## 1. Introduction

Crohn’s disease is a chronic inflammation of the gastrointestinal (GI) tract that arises from complicated and unclear interactions between genetic predispositions and environmental factors [1,2]. CD may affect any part of the GI tract; however, in most cases, it involves the ileum, colon, or both [2]. Over the last 50 years, the increasing prevalence of CD has been observed, with the highest incidence rate recorded in northern Europe, the United Kingdom, and North America [2]. Initially, CD manifests itself between the ages 15 and 39, with a second peak between 50 and 70 [2,3]. Gender influence was found to be different in various demographics [2].

Patients usually suffer from chronic diarrhea [1,2] for more than 4 weeks, which is a major symptom of the CD progression [2]. Furthermore, abdominal pain (70%), weight loss (60%) [2], malnutrition [1,3], and blood and mucus present in the stool (40–50%) [2] are also commonly occurring symptoms of CD.

Crohn’s disease exerts a strong impact on the quality of patients’ lives, especially regarding lifestyle and diet. For instance, patients who suffer from CD avoid consuming so-called “trigger food” and follow strict dietary recommendations [1,2]. Of crucial importance is the regular taking of prescribed medication. Moreover, pain and fatigue might occur during acute flares of Crohn’s disease, which both become an impediment to daily activities resulting in absence from employment or school, as reported by patients [2].

Initially, pharmacological disease management is undertaken to suppress the inflammatory response [2] and reduce the frequent occurrence of diarrhea, leading to a significant loss of nutrients [4]. For induction of remission, corticosteroids, budesonide, mesalazine, or 5-aminosalicylates are commonly used. The anti-tumor necrosis factor (TNF) immunosuppressive therapies are involved in patients with resistance to conventional therapy [2]. Surgical resection is necessary for most patients, usually within 10 years of their diagnosis, as most cases become medically exhausted. The surgery might improve the quality of life and manage the progressive and/or significant loss of weight, intestinal obstruction reoccurrences, and the risk of septic complications such as perforations [2].

However, in patients with changes or abnormalities in the metabolism of saccharides [5,6], essential amino acids [6], and lipids [5,6,7], wounds and tissue healing might become difficult. Malnutrition and electrolyte imbalance are two risk factors for the occurrence of severe post-operative complications in CD patients [8]. In this study, the influence of the operational intervention on blood plasma metabolome was analyzed in patients with advanced CD. A well-developed GC/MS-based approach was utilized to determine fluctuations in plasma metabolites upon the surgical resection.

## 2. Results

In the first step, clinical parameters were compared pre- and post-operation for all patients, and no differences were observed of statistical significance, considering FDR corrected *p*-values. Increased C-reactive protein (CRP) levels were found in all patients after the surgery and in the case of five patients before surgery. In CD, a strong CRP response may occur [9]. In general, the clinical parameters indicate proper clinical management prior- and post-surgery. A summary of all clinical data is provided in Table 1 and Table 2.

The GC/MS-based approach enabled the identification of 155 metabolites in blood plasma samples taken from patients with Crohn’s disease pre- and three days after the surgical treatment. Among them, 108 compounds were determined in most samples and subjected to statistical analysis. A table of the identified compounds and an example chromatogram of a patient plasma sample are shown in Table S1 and Figure S1 in the Supplementary Material.

The paired *t*-test was applied, followed by the correction for multiple comparisons, to evaluate the statistical significance of fluctuations in levels of individual metabolites pre- and post-surgery. A statistically significant (*p*-value < 0.05) increase was found in levels of 11 metabolites and a decrease in the abundance of two compounds in blood plasma after the surgery compared to the state prior to surgery. However, only five metabolites remained statistically significant after the additional FDR correction (FDR corrected *p*-value < 0.05), including glycerol 3-phosphate and four monoacylglycerol species (MG). Furthermore, ribonic acid was determined in the blood plasma of only two patients after the surgery (2/7 collected samples).

In the blood plasma of patients after the surgery, a substantial accumulation of glycerol 3-phosphate was observed (FC = 4.33), α-tocopherol (FC = 3.40), and four monoacylglycerols. Interestingly, MG species, which are two pairs of isomers, showed a similar increase, i.e., 2.79- and 2.74-fold for 1- and 2-palmityloylglycerols; 2.63- and 2.35-fold for 1- and 2-stearolyglycerols, respectively. Nearly two times higher levels of three amino acids were found, including one endogenous amino acid L-cysteine and two exogenous: L-lysine and L-methionine. Additionally, succinic acid was 2.5 times higher, and benzoic acid showed a 1.57-fold increase.

In turn, a significant 11-fold decrease in abundance of galactopyranose and three times lower levels of 3,4-dihydroxybutanoic acid were determined in the plasma of patients post-surgery. Since ribonic acid was registered in two plasma samples collected post-surgery, it could also be considered a down-regulated metabolite.

**Figure 1:**
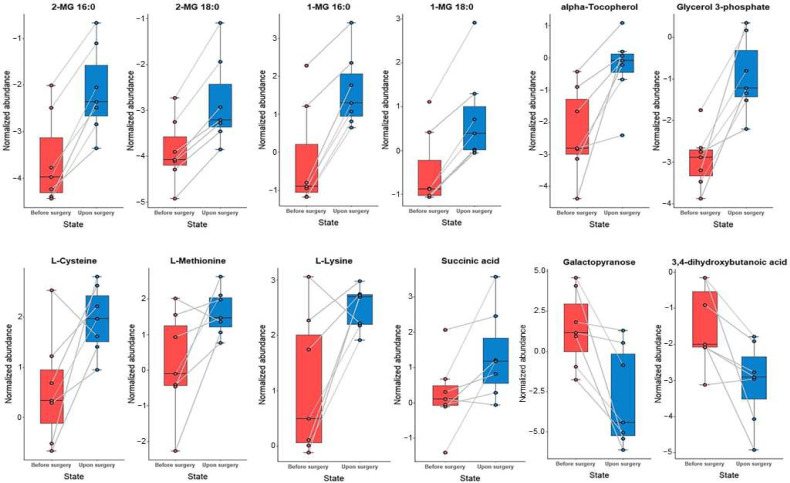
Box plots illustrating changes in levels of selected metabolites before and after the surgery.

**Figure 2:**
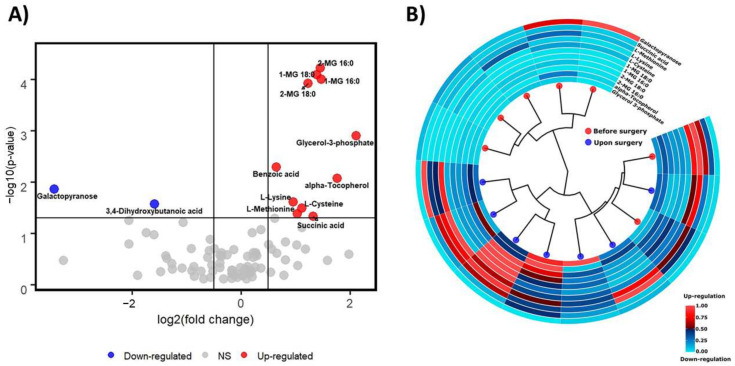
(**A**) Volcano plot shows metabolites with the raw *p*-value < 0.05 and simultaneously log2 (fold change) above 0.5; (**B**) Circular dendrogram and heatmap created using the set of biologically relevant metabolites. Further, 0–1 scaling was applied to normalize the abundance of individual metabolites in patients’ plasma prior- and post-surgery.

The complete summary of the results and box plots illustrating the differences in levels of individual metabolites pre- and post-surgery are presented in Table 3, Figure 1 and Figure 2.

**Figure 3:**
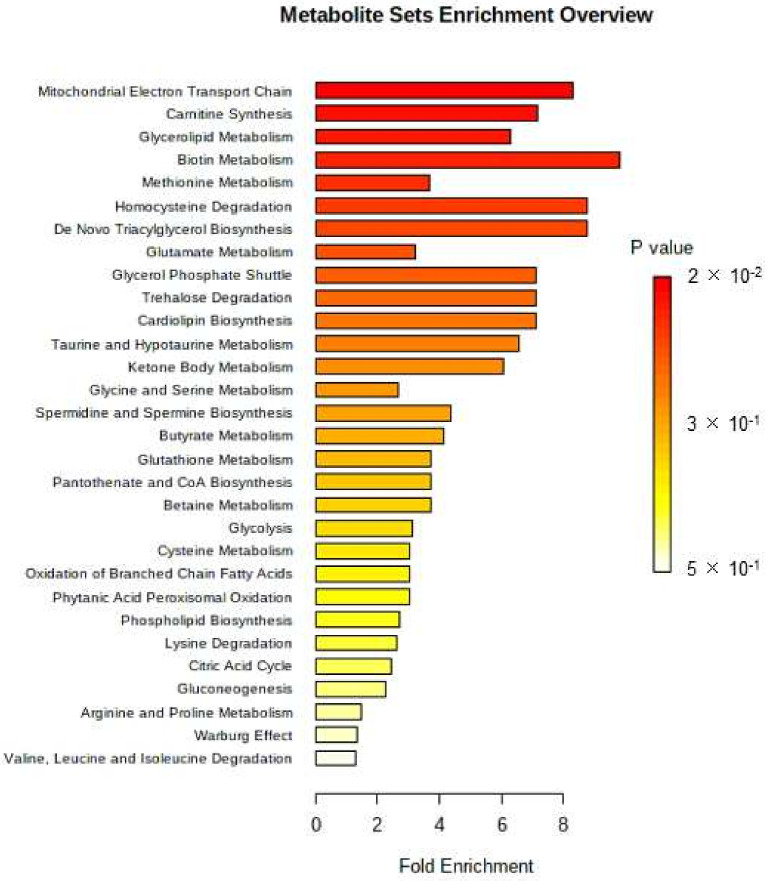
Results of the Metabolite Set Enrichment Analysis with Fold Enrichment higher than 4.

In the next step, using metabolites selected in the statistical analysis, the Metabolite Set Enrichment Analysis was applied to suggest biological pathways of potential importance. The ORA enrichment analysis was used to assess if particular sets of metabolites were represented more than expected by chance within the provided compound list. Three metabolite sets were found with *p*-value < 0.05, namely mitochondrial electron transport chain (2/19, expected 0.26), carnitine synthesis (2/22, expected 0.301), and glycerolipid metabolism (2/25, expected 0.352), which were associated with up-regulated glycerol 3-phosphate, succinic acid, L-Lysine and 2-MG 16:0 (Figure 3).

## 3. Discussion

This study’s main goal was to compare plasma metabolites’ profiles before and after the ileo-colic region resection in the case of seven patients with active and advanced Lesniowski-Crohn’s disease.

In general, results of MSEA suggest activation of mitochondrial pathways and related processes. One of the major metabolic goals after a strong trauma, such as a surgical injury, is an intensive endogenous synthesis of glucose (gluconeogenesis) [10,11]. The enhanced glucose production ensures substrate supply to compromised tissues and cells, in which mitochondrial respiration is not (yet) possible [10]. However, 80–90% of the energy for gluconeogenesis is provided by free fatty acids (FFAs) oxidation in mitochondria [10,11]. FFAs are obtained through triacylglycerols (TG) lipolysis. TG species undergo sequential hydrolysis, and fatty acids are removed preferentially from the sn-1 or sn-3 positions, finally resulting in the obtainment of 2-MG [12]. The isomerization of 2-MG to 1-MG enables further hydrolysis of 1-MG species to free fatty acids and glycerol [13]. Therefore, the increased levels of 1- and 2-MG might indicate lipolysis initiation, followed by the FFAs beta-oxidation. Noteworthy in this respect is that we observed higher levels of L-lysine and succinic acid in blood plasma upon surgery linked to carnitine synthesis regarding MSEA results. Carnitine actively participates in the transport of fatty acids from the cell’s cytoplasm through the mitochondrial membrane to enable their β-oxidation [12].

A high energy demand post-surgery could also be reflected in a nearly 11-fold decrease in plasma galactopyranose levels. Galactose is metabolized mainly in the Leloir pathway to glucose 6-phosphate, which subsequently enters pathways of hexoses’ breakdown, e.g., glycolysis [14].

Furthermore, increased glycerol 3-phosphate and succinic acid levels were linked to the activation of the mitochondrial electron transport chain (ETC) and glycerol-3-phosphate shuttle, based on the MSEA. The ETC drives the generation of ATP via the electrochemical gradient of protons, caused by the energy obtained in redox reactions, in which glycerol 3-phosphate actively participates [15].

Higher abundances of L-cysteine, L-methionine, and α-tocopherol (vitamin E) were also observed upon surgery. L-methionine is the precursor of endogenous L-cysteine, which in turn is converted to glutathione, an essential antioxidant [16,17]. Regarding the MSEA, among overrepresented pathways were methionine, cysteine, and glutathione metabolism, as well as homocysteine degradation. α-Tocopherol (vitamin E) is also a crucial antioxidant that essentially prevents lipid oxidation. As intense oxidation processes are suspected to occur in mitochondria after the surgical trauma, an increased risk of reactive oxygen species (ROS) leakage occurs. The ROS might cause significant damage to cellular structures and, in this way, disrupt their functions [18]. Thus, higher plasma levels of these compounds might be related to the maintenance of oxidative stability of mitochondrial and cellular membranes.

Changes in metabolome and lipidome in patients with inflammatory bowel diseases are intensively investigated using different biological matrices (serum, urine, tissues) and analytical techniques (LC/MS, GC/MS, NMR). Usually, metabolomes or lipidomes of patients with Crohn’s disease or ulcerative colitis (UC) are compared to healthy volunteers to find potentially useful biomarkers for clinical purposes. Daniluk et al. presented in their LC/MS-based study the downregulation of serum phospholipids in patients with Crohn’s disease (*n* = 9) and ulcerative colitis (*n* = 10) compared to healthy volunteers (*n* = 10) and a significant upregulation of LacCer 18:0/16:0 in patients with Crohn’s disease compared to healthy controls. LacCer 18:0/16:0 was also successfully tested alone and with other serum inflammatory markers as a potential marker of Crohn’s disease, allowing differentiation from ulcerative colitis. The subsequent pathway analysis indicated possible alterations in the glycerophospholipids metabolism and sphingolipid metabolism in patients with Crohn’s disease [7]. Stephens et al. performed urine sample analysis from patients with inflammatory bowel diseases and healthy controls, using nuclear magnetic resonance (NMR) spectroscopy combined with targeted profiling techniques. As a result, the decrease was observed in TCA cycle intermediates (succinate and citrate), amino acids (asparagine, lysine, histidine, and 1-methylhistidine), gut microflora metabolites (methanol, formate, hippurate, acetate, and methylamine), trigonelline, creatine, urea, and taurine, in patients with inflammatory bowel diseases compared to controls [19]. Stephens et al. also compared their results to those obtained by Williams et al. Both studies had similar concepts and outcomes. Moreover, Williams et al. did not find any differences between patients with CD who underwent bowel resections and those who had not [20]. Pierre Martin et al. investigated urine samples from 21 pediatric patients with IBD and 27 healthy children using NMR spectroscopy and reported alterations in metabolites related to central energy metabolism, amino acids, and metabolites belonging to gut microbial metabolic pathways [21]. In turn, Dawiskiba et al. performed an NMR-based metabolomics analysis of serum and plasma samples collected from 24 patients with ulcerative colitis, 19 patients with Crohn’s disease (CD), and 17 healthy controls. The results of their work correspond well with other reports and include an increase in serum leucine, isoleucine, 3-hydroxybutyric acid, N-acetylated compounds, acetoacetate, glycine, phenylalanine, and lactate levels, a decrease in serum levels of creatine, dimethyl sulfone, histidine, choline, and its derivatives, and a decrease in urine levels of citrate, hippurate, trigonelline, taurine, succinate and 2-hydroxyisobutyrate in patients with IBD compared to healthy controls [22]. In these studies, UC and CD caused similar alterations in metabolic profiles and distinguishing CD from UC was difficult or not possible. Only Stephens et al. reported the possibility of distinguishing between CD and UC but only when CD patients who underwent surgical intervention or were on anti-TNF-α treatment were retained in the dataset for the comparison [20]. Ooi et al. presented GC/MS-based results of metabolites profiling in biopsies from 22 UC patients and serum samples from UC patients (*n* = 13), CD patients (*n* = 21), and healthy volunteers (*n* = 17). In the case of tissue samples, 16 amino acids and 6 metabolites involved in the TCA cycle were significantly decreased in UC patients. Analysis of serum samples from CD patients revealed the upregulation of alanine, aspartic acid, glycine, methionine, proline, fumaric acid, malic acid, and succinic acid and the downregulation of glutamine, histidine, and tryptophan when compared to healthy volunteers. The differences were also shown between UC patients’ and CD patients’ metabolomes [23]. However, comparing the reported outcomes with the results presented in this manuscript can be difficult. The study did not include a healthy control group, and it was focused on investing plasma alterations appearing upon the surgical intervention. Therefore, the outcomes are likely related to high energy demand in post-surgery recovery. It is noteworthy that metabolomics can be used for predicting the therapeutic response in IBD [24]. However, the observed alterations in the metabolome may depend on the type of therapy. Thus far, the application of anti-tumor necrosis factor, vedolizumab, Infliximab, exclusive enteral nutrition, and fecal microbiota transplantation has been widely investigated [24]. For the follow-up study, more different types of samples should be collected (plasma, urine, feces) at more time points to widely verify the current observations and evaluate long-time outcomes of ileo-colic region resection.

In summary, three days after the surgery in the blood plasma of patients, the characteristic increase was found in levels of saturated monoacylglycerols, lysine, methionine, cysteine, glycerol-3-phosphate, α-tocopherol, and succinic acid, and a significant decrease in galactose level compared to the state before surgery. Based on the Metabolite Set Enrichment Analysis, the changes observed in the metabolome could be linked to the mitochondrial processes’ activation, reflecting a high energy demand in the post-surgery recovery. Most of these metabolites are simultaneously essential nutrients supplied from a daily diet, e.g., monoacylglycerols or galactose, or only from the diet, e.g., exogenous amino acids: lysine and methionine, or tocopherol. Hence, the results are also consistent with critical aspects of perioperative care, highlighting the possible role of fast re-establishment of oral feeding after surgery and avoidance of pre-operative fasting in the enhanced recovery [25].

## 4. Materials and Methods

### 4.1. Characteristics of Patients

Blood samples were taken and originally anonymized by the Department of Gen-eral, Oncological and Digestive Tract Surgery of the Medical Centre of Postgraduate Education, Orłowski Hospital, Warsaw, and secondary anonymized by Department of Biochemistry and Clinical Chemistry, Medical University of Warsaw. Serum samples were obtained from 7 patients with active Crohn’s disease (CD) according to the Harvey-Bradshaw score (the active form of the disease defined as ≥5) [26], qualified for the ileo-colic region resection. A standard low-fat diet with limited fiber content and increased protein content was administered to patients in the pre-operative period. On the first day after surgery, intravenous fluid and electrolyte infusion was used. On the second day after surgery, enteral nutrition began, initially in liquid form and from the third day in the form of solid foods. The use of human blood subjects for this study was approved by the Medical Centre of Postgraduate Education Ethical Committee. The general characteristics of patients are presented in Table 4. In the study were enrolled 3 men (36 ± 10 years old) and 4 women (42 ± 17 years old).

### 4.2. Blood Collection

Two consecutive blood plasma samples were collected from patients (*n* = 7), pre- and post-surgery, respectively. Blood was collected in the morning, in fasting state, on the day before surgery, and on the third day after surgery. Blood samples were centrifuged for 10 min at a speed of 1500× *g* at 4 °C to obtain plasma. Finally, samples were portioned into 1.5 mL tubes adapted for low temperatures, frozen and transported on dry ice, and stored at −80 °C for further analysis.

### 4.3. Metabolites Extraction and Derivatization

Low molecular weight metabolites were extracted from 30 μL of blood plasma with 1 mL of ice-cold mixture of acetonitrile, isopropanol, and water (3:3:2 *v*/*v*/*v*) [27]. Briefly, all samples were homogenized for ~10 s, then shaken at 4 °C for 5 min, and finally centrifuged at 13,000 rcf (2 min, at 4 °C). Supernatants were removed and split into two 450 μL portions: the first one was subjected to the analysis and the second one was a backup sample. Extraction solvents were evaporated at 30 °C in a rotary vacuum concentrator (Eppendorf), and dry precipitates were subsequently derivatized. A two-step derivatization procedure was performed. First, 10 μL of methoxyamine hydrochloride solution was used (20 mg/mL in dry pyridine), and samples were kept at 37 °C for 90 min. In the second step, 90 μL of N-methyl-N-trimethylsilyltrifluoroacetamide (MSTFA) was applied for silylation, and the reaction was continued for another 30 min at 37 °C. All samples, QCs (pools and standard mixtures), and blanks were centrifuged, transferred to glass chromatographic vials, and then subjected to the GC/MS analysis.

### 4.4. GC/MS System and Spectra Processing

The GC/MS system consisted of Agilent 7890B gas chromatograph with the S/SL inlet, connected to the Pegasus BT time-of-flight mass spectrometer (LECO Corporation). For metabolites separation, the standard Restek Rxi-5MS fused-silica capillary column of a low-polarity bonded phase was selected in following dimensions: 30 m length, 0.25 mm ID, and 0.25 μm film thickness. The constant flow of helium was set to 1 mL/min, and 0.5 μL of each sample was injected in splitless mode at 280 °C. The inlet purge flow rate was 40 mL/min, and the septum purge flow 3 mL/min, respectively; the inlet was purged 70 s after injection. The GC oven temperature program was as follows: 1 min at 70 °C, raised subsequently by 12 °C/min to 300 °C, and held for 14 min (total run time 34 min and 10 s, 336 s of solvent delay). The transfer line temperature was kept at 300 °C, and the ion source temperature was 250 °C. EI-MS spectra were recorded in the *m*/*z* range 50–650, at the acquisition rate of 12 spectra/second, and the standard electron ionization energy of 70 eV was used. The GC/MS system suitability was verified within a series of autotunes and controlled during the sequence using quality control samples and blanks. The obtained GC/MS profiles were exported as ANDII MS files and transferred from the ChromaTOF software for Pegasus BT (ver. 5.32) to ChromaTOF (ver. 4.51.6.0) with the stat compare module for data processing. The automatic peak detection, deconvolution, retention index calculation, and library search were performed subsequently. Based on the analysis of alkanes mixture (C10–C36), retention indices (RI) were estimated to improve identification results and to correct retention times (RT). For the identification of the compounds, the Mainlib and Fiehn libraries were used; quality filter assumed similarity index (SI) >700. The unique quantification mass for each compound was defined and used to obtain accurate peak areas for the statistical comparison. Unknown compounds and impurities (i.e., plasticizers, column bleeds, alkanes, siloxanes) were removed from the obtained table of data. The identifications were subsequently compared to those obtained from the MS Dial software (v 4.80) [28]. The calculated values of RI were compared to theoretical values obtained from the Kovats RI library for MS Dial (9062 unique compounds), NIST Chemistry WebBook, PubChem database, and Human Metabolome Database.

### 4.5. Statistical and Bioinformatic Analysis

Peak areas were derived from predefined and unique for each metabolite quantification mass. Features with >50% missing values were removed, and the k-nearest neighbors approach was used to estimate the remaining missing values. Data were additionally filtered based on relative standard deviation (RSD = SD/mean). Samples were normalized to the sum of all detected signals and log-transformed. Box plots were created using the ggplot2 and ggpubr packages. The lower and upper hinges represent 25% quantile and 75% quantile, respectively, and the middle line median (50% quantile). The lower whisker is the smallest observation greater than or equal to the lower hinge—1.5·IQR (interquartile range)—and the upper whisker is the largest observation less than or equal to the upper hinge + 1.5·IQR. The statistical comparison of normalized peak areas was performed in the R environment (rstatix package). The significance of differences in plasma metabolites’ abundances before and after the surgical treatment was assessed based on the paired *t*-test. Then, the Benjamini–Hochberg FDR approach was applied to all obtained *p*-values for the multiple testing correction. The fold change (FC) was calculated for all metabolites by dividing the abundance post-treatment by the abundance prior treatment. Obtained *p*-values and fold changes were then used to create the volcano plot (EnhancedVolcano package). The circular dendrogram surrounded by the heatmap presenting the biologically most interesting metabolites was generated using the ggtree package. Metabolites selected in the statistical test were listed, and input was created for the Metabolite Set Enrichment Analysis (MSEA, available at https://www.metaboanalyst.ca/MetaboAnalyst/upload/EnrichUploadView.xhtml (accessed on 21 November 2020)), which was employed to facilitate the identification of biologically meaningful patterns [29]. The statistical significance of the obtained overrepresentation (ORA) was estimated using the hypergeometric test.

## Figures and Tables

**Table 1:** Summary of patients’ clinical data.

Patient ID	Protein [g/dL]				Electrolytes										Glucose [mg/dL]		CRP [mg/L]		Creatinine [mg/dL]		AST [U/L]		ALT [U/L]		Urea [mg/dL]		Coagulation System [s]					
	Total		Albumins		Na [mM]		K [mM]		Ca [mg/dL]		Mg [mg/dL]		P_inorg._ [mg/dL]														PT		INR		APTT	
	B	A	B	A	B	A	B	A	B	A	B	A	B	A	B	A	B	A	B	A	B	A	B	A	B	A	B	A	B	A	B	A
1	5.0	5.1	2.9	2.3	143	139	4.5	4.4	8.5	8.2	2.3	2.6	3.5	3.8	97	71	52	95	0.80	0.82	18	20	13	26	46	42	11.9	12.2	0.99	1.01	30	32
2	6.2	5.4	3.1	3.1	138	137	4.4	4.5	7.6	8.3	1.7	2.1	1.8	3.5	75	84	193	225	0.58	0.65	no data	no data	no data	no data	8	16	13.7	15.8	1.14	1.31	33	40
3	6.7	6.4	3.0	2.7	140	144	4.0	3.7	8.2	7.6	1.6	2.1	3.3	3.7	106	83	85	132	0.88	0.78	15	17	23	42	18	41	13.3	15.8	1.17	1.41	40	40
4	5.7	4.9	2.8	2.2	140	139	4.0	2.7	7.8	7.9	1.8	1.9	3.2	2.9	113	107	2	126	0.73	0.63	16	22	34	29	15	24	11.2	10.7	0.93	0.99	30	33
5	5.9	5.7	2.8	2.8	140	142	3.3	5.0	8.1	8.8	2.0	2.8	3.1	3.0	101	82	76	29	0.85	0.97	44	52	39	66	20	19	12.3	11.8	1.10	1.04	32	28
6	6.8	7.0	4.2	3.8	140	138	4.1	4.3	8.8	8.9	1.7	1.8	2.4	3.3	60	99	2	84	1.15	0.93	21	33	15	36	25	26	11.3	11.2	0.99	0.94	29	31
7	6.2	6.2	2.8	3.0	139	141	4.8	4.6	8.1	8.4	2.0	1.6	2.9	2.7	76	91	181	100	0.65	0.69	16	11	23	35	14	26	11.5	11.9	1.02	1.06	33	33

B—before surgery; A—after surgery.

**Table 2:** Statistical analysis of clinical characteristics of patients in the study.

Clinical Parameter	Before Surgery	After Surgery	Laboratory Norms	Statistic	*p* Value	FDR Adjusted *p* Value	Remarks
Total protein [g/dL]	6.1 ± 0.5	5.8 ± 0.8	6.0–8.3	−1.56	0.177	0.40	ns
Albumin [g/dL]	3.1 ± 0.5	2.8 ± 0.5	3.4–5.4	−2.04	0.09	0.40	ns
Total cholesterol [mg/dL]	121.3 ± 30.6	157.0 ± 19.1	<200	-	-	-	^1^
TG [mg/dL]	76.0 ± 33.8	118.7 ± 18.6	<150	-	-	-	^1^
Na [mM]	140.0 ± 1.5	140.0 ± 2.4	135–147	0.00	1.00	1.00	ns
K [mM]	4.2 ± 0.5	4.2 ± 0.8	3.0–5.5	0.04	0.97	1.00	ns
Ca [mg/dL]	8.1 ± 0.4	8.3 ± 0.5	8.6–10.3	0.84	0.43	0.58	ns
Mg [mg/dL]	1.9 ± 0.2	2.1 ± 0.4	1.8–2.4	1.80	0.12	0.40	ns
Phosphorus (inorganic) [mg/dL]	2.9 ± 0.6	3.3 ± 0.4	2.5–4.5	1.43	0.20	0.40	ns
Glucose [mg/dL]	89.7 ± 19.5	88.1 ± 12.0	72.0–99.0	−0.17	0.87	0.99	ns
CRP [mg/L]	84.3 ± 77.2	113.0 ± 59.7	<3.0	1.07	0.33	0.47	ns
Creatinine [mg/dL]	0.81 ± 0.19	0.78 ± 0.13	0.84–1.21	−0.54	0.61	0.75	ns
AST [U/L]	21.7 ± 11.1	25.8 ± 14.7	8.0–48.0	1.74	0.14	0.40	ns, ^2^
ALT [U/L]	24.5 ± 10.3	39.0 ± 14.4	7.0–55.0	3.22	0.02	0.37	p, ns, ^2^
Urea [mg/dL]	20.9 ± 12.3	27.7 ± 10.1	7.0–20.0	1.97	0.10	0.40	ns
PT [s]	12.2 ± 1.0	12.7 ± 2.1	11.0–13.5	1.22	0.27	0.47	ns
INR [s]	1.0 ± 0.1	1.1 ± 0.2	0.8–1.1	1.44	0.20	0.40	ns
APTT [s]	32.4 ± 3.5	33.8 ± 4.6	30.0–40.0	1.10	0.31	0.47	ns

ns—not significant after FDR correction (FDR corrected *p*-value < 0.05); ^1^ Total cholesterol and TG were measured for 3 patients only; ^2^ AST and ALT were measured for 6 patients; p—raw *p*-value significant (*p*-value < 0.05).

**Table 3:** Statistical analysis of clinical characteristics of patients in the study.

Metabolite	Class of Metabolites	RT [min]	Mean [a.u.]	C.V.	Mean [a.u.]	C.V.	After/Before Ratio	*p* Value	FDR *p* Value
Glycerol-3-phosphate	Phosphoric acid deriv.	13.12	0.144	0.518	0.622	0.657	4.33	0.00125	0.029
α-Tocopherol	Lipids	23.26	0.291	0.886	0.991	0.599	3.40	0.00845	0.140
1-Monopalmitoylglycerol	Lipids	19.17	1.385	1.209	3.858	0.833	2.79	0.00010	0.004
2-Monopalmitoylglycerol	Lipids	18.9	0.102	0.786	0.279	0.703	2.74	0.00006	0.004
1-Monostearoylglycerol	Lipids	20.34	0.864	0.751	2.272	1.045	2.63	0.00008	0.004
Succinic acid	Carboxylic acids	8.46	1.482	0.841	3.705	1.055	2.50	0.04682	0.362
2-Monostearoylglycerol	Lipids	20.1	0.075	0.534	0.176	0.814	2.35	0.00012	0.004
L-Cysteine	Amino acids	11.1	1.938	0.929	4.214	0.444	2.17	0.03241	0.342
L-Methionine	Amino acids	10.74	1.645	0.848	3.364	0.462	2.05	0.04194	0.362
L-Lysine	Amino acids	14.36	2.989	0.930	5.814	0.257	1.95	0.02451	0.315
Benzoic acid **	Carboxylic acids	7.81	0.922	0.560	1.449	0.361	1.57	0.00511	0.099
3,4-Dihydroxybutanoic acid	Carboxylic acids	9.8	0.452	0.728	0.151	0.637	0.33	0.02717	0.315
Galactopyranose	Sugars	14.32	7.096	1.336	0.661	1.462	0.09	0.01396	0.202
Ribonic acid *	Sugar deriv.	13.35	7/7	2/7	NA				

* Metabolite was not determined in the blood plasma of 5 patients after the surgery. ** Identification uncertain. NA—not applied.

**Table 4:** General patients characteristics.

Patient ID	Gender	Age	Disease State
1	M	42	active
2	F	29	active
3	M	43	active
4	F	52	active
5	F	60	active
6	M	24	active
7	F	26	active

## Data Availability

The data presented in this study are available on request from the corresponding author. The data are not publicly available due to restrictions on privacy.

## Supplementary Materials

The following supporting information can be downloaded at: https://www.mdpi.com/article/10.3390/metabo12060529/s1, Table S1: Identified compounds. Figure S1: GC-MS chromatogram of blood plasma sample of patient.
