# Supplementary figures and images for: Alterations in Blood Plasma Metabolome of Patients with Lesniowski-Crohn’s Disease Shortly after Surgical Treatment—Pilot Study
Source: Metabolites. 2022 Jun 8;12(6):529. doi: 10.3390/metabo12060529 (PMC9228040; doi:10.3390/metabo12060529)

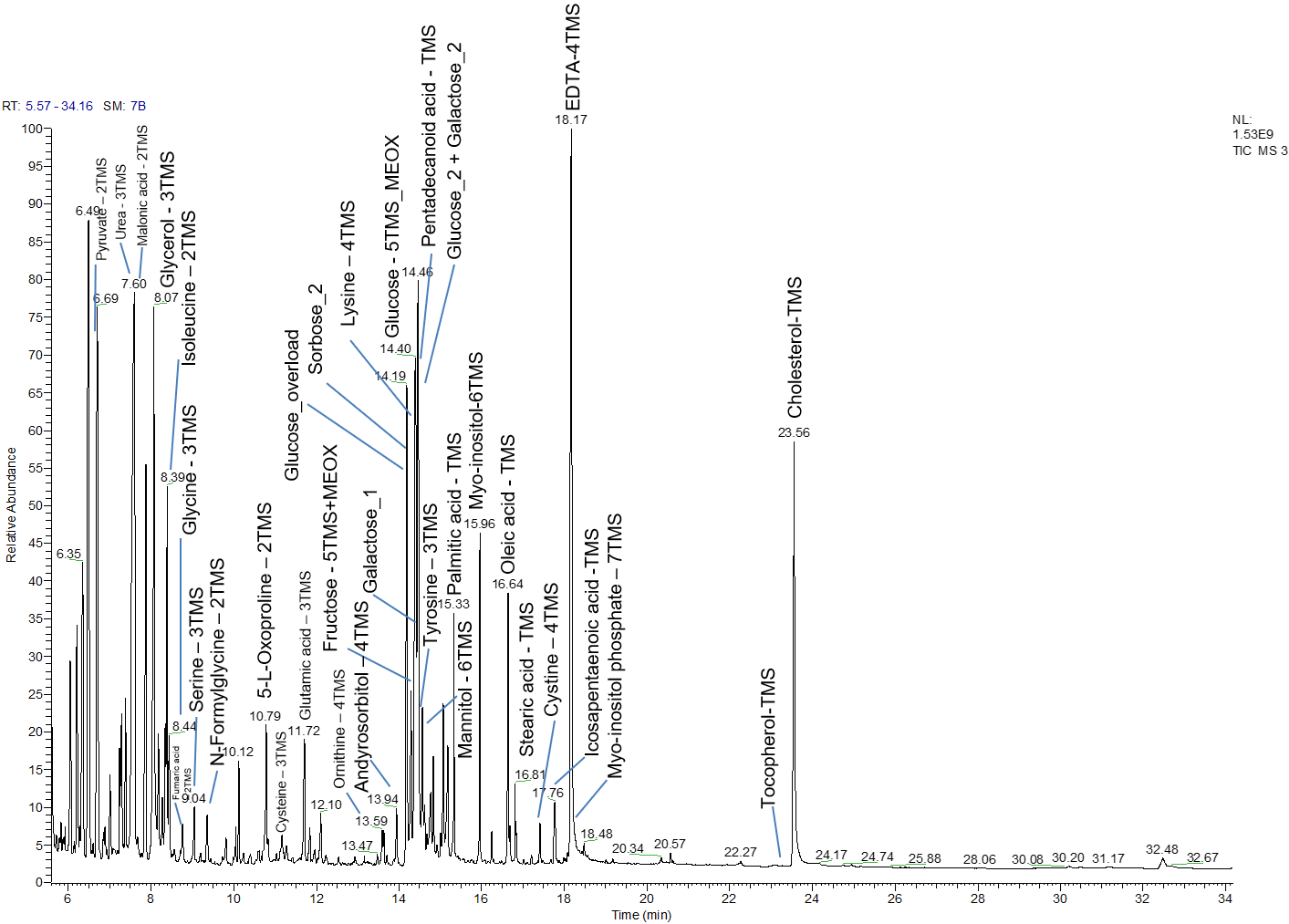

Supplement: Supplementary file 1 [file metabolites-12-00529-s001.zip › Figure S1.tif]
